# Acute pancreatitis related to therapeutic dosing with colchicine: a case report

**DOI:** 10.1186/1752-1947-1-64

**Published:** 2007-08-12

**Authors:** Joseph Yuk Sang Ting

**Affiliations:** 1 Department of Emergency Medicine, Mater Adult Hospital, Raymond Tce, South Brisbane 4101, Australia

## Abstract

**Background:**

Colchicine is used in the treatment and prophylaxis of gout. It possesses a narrow therapeutic window, frequently resulting in dose-limiting gastrointestinal side-effects such as diarrhoea and emesis. As colchicine is a cellular anti-mitotic agent, the most serious effects include myelosuppression, myoneuropathy and multiple organ failure. This occurs with intentional overdose or with therapeutic dosing in patients with reduced clearance of colchicine due to pre-existing renal or hepatic impairment. Acute pancreatitis has rarely been reported, and only in association with severe colchicine overdose accompanied by multi-organ failure.

**Case presentation:**

We report a case of acute pancreatitis without other organ toxicity related to recent commencement of colchicine for acute gout, occurring in an elderly male with pre-existing renal impairment.

**Conclusion:**

1) Colchicine should be used with care in elderly patients or patients with impaired renal function.

2) Aside from myelosuppression, myoneuropathy and multiple organ failure, colchicine may now be associated with acute pancreatitis even with therapeutic dosing; this has not previously being reported.

## Background

Colchicine is frequently used to treat and prevent recurrence of acute gout [1] but has a narrow therapeutic window, with dose-limiting gastrointestinal side-effects such as diarrhoea and vomiting [2]. Colchicine toxicity relates to its cellular anti-mitotic action and preferentially affects tissues that have a rapid turnover [3], leading to early gastrointestinal failure, myelosuppression and ultimately multi-organ failure [1,2]. Colchicine in intentional overdose is difficult to treat and frequently lethal [4]. Acute pancreatitis related to colchicine has rarely been reported, and only in the context of severe toxicity [5-7].

## Case presentation

A 79 year old man presented to the Emergency Department with severe epigastric pain, nausea, vomiting without hematemesis, diarrhoea and anorexia. He has a history of red cell and platelet transfusion dependent myelofibrosis, iron overload due to multiple red cell transfusion, chronic renal failure, ischemic heart disease, left ventricular systolic dysfunction, cerebrovascular disease, peripheral vascular disease, hypertension, and hyperlipidemia. His regular medications include diltiazem, valacyclovir, nicorandil, bisoprolol, deferosirox, frusemide and glyceryl trinitrate patches. Aside from the recent addition of colchicine, his medications are unchanged. The patient denied using herbal or over the counter products. He was independent with activities of daily living, was a life-long non-alcohol drinker and stopped smoking decades ago. There was no history of abdominal trauma.

The patient was alert and orientated with blood pressure 100/48 mm Hg, pulse 66/min regular, respiratory rate 20/min, SaO2 is 97% on room air and temperature 36.8°C. There was marked epigastric tenderness without abdominal distension. Normal bowel sounds are present. No free subdiaphragmatic gas was visible on erect chest X-ray.

Several blood investigations were performed; serum lipase is elevated at 859 (reference range, RR 25–300 U/L). Serum urate is 0.92 (RR 0.15–0.5 mmol/L), albumin-corrected calcium 2.38 (RR 2.15–2.6 mmol/L), sodium 136 (RR 135–145 mmol/L) and potassium 5.0 (RR 3.2–4.5 mmol/L). There was a mild deterioration of serum creatinine to 332 (RR 70–120 umol/L) and urea 38.9 (RR 3.0–8.0 mmol/L). Venous blood gases showed pH of 7.24 (RR 7.35–7.45), bicarbonate 18 (RR 22–27 mmol/L), base deficit -8.9 (RR -3.0 to +3.0 mmol/L) and lactate 1.0 (RR 0.2–2.0 mmol/L). Serum triglyceride level was 2.0 (RR 0.5–2.0 mmol/L). Liver function tests were deranged: aspartate transaminase 206 (RR 10–45 U/L), alanine transaminase 269 (RR 5–45 U/L), gamma glutamyl transaminase 145 (RR 10–70 U/L), alkaline phosphatase 209 (RR 40–110 U/L) and total bilirubin 9 (RR <20 micromoles/L). Prothrombin time was 16.1 (RR 11–16 seconds), INR 1.3 and APTT 33.4 (RR 22–35 seconds). Haematology parameters were unchanged from previous results, with haemoglobin 118 (RR 130–180 g/L), haematocrit 0.352 (RR 0.40–0.54), white cell count 7.8 (RR 4.5–11.0 × 10^9^/L) and platelets 34 (RR 150–400 × 10^9^/L).

The patient recovered over 48 hours with clear oral fluids and supportive medical treatment including intravenous fluids and analgesia as required. This coincided with declining lipase levels. Myelosuppression ascribable to colchicine did not occur.

An abdominal ultrasound demonstrated normal liver texture, oedematous pancreas, and multiple small incidental calculi within a normal walled gallbladder, no hepatobiliary ductal dilatation or pericholecystic fluid, normal pancreatic duct, enlarged spleen and multiple bilateral renal cysts without hydronephrosis.

## Discussion and conclusion

The incidence of acute pancreatitis varies in different countries and depends on cause, with the estimated incidence in England being 5.4/100 000 per year; in the United States it is 79.8/100 000 per year [14]. Precipitants of acute pancreatitis are extensive and wide ranging [10]; the most frequent causes are gallstones (30–60%) and alcohol (15–30%) [8,14]. In 20% of cases, the cause remains unidentified [8]. Drugs are implicated in only 2–5% of cases, either by a hypersensitivity reaction or the generation of a toxic metabolite [14], although it is frequently difficult to prove causality [9].

Identifying the underlying cause of acute pancreatitis allows avoidance or treatment of the precipitant and improves chances of recovery [10]. This patient sustained mild and self-limited acute pancreatitis associated with recent commencement of colchicine for gout, which has not previously been reported. However, comorbidities implicated in acute pancreatitis make a trigger or co-factor role for colchicine more likely [9], rather than colchicine being the sole aetiological agent. In this case, microlithiasis [8], chronic renal failure [10,11,14] and frusemide [10] may have set the scene for acute pancreatitis precipitated by colchicine. With a normal bilirubin level, however, the patient had liver function tests which were more consistent with a hepatitic rather than an obstructive enzymosis. Furthermore, he did not have hypercalcaemia or hypertriglyceridaemia, metabolic factors well known to contribute to pancreatic inflammation [8,14].

There was no evidence of acute seroconversion to hepatitis A, B, C viruses; Epstein-Barr virus, Cytomegalovirus and herpes simplex 1 and 2 viruses. As such, acute viral hepatitis or pancreatitis was unlikely. Non-drug aetiologies for pancreatitis in this patient (renal failure, microlithiasis, ongoing use of frusemide) remained; despite this, rapid clinical recovery occurred with withdrawal of colchicine. This renders colchicine the most eminent association to pancreatitis in this case.

Sole attribution for acute pancreatitis to a single drug remains difficult due to high rates of concurrent contributory diseases in acute pancreatitis [9]. Aside from frusemide, of which there had been no recent dose escalation, none of this patient's other prescribed medications have been reported to increase risk of acute pancreatitis [10,14]. Nitrates have been known to reduce pancreatitis pain and relapse [13], with diltiazem improving survival in rat models of acute pancreatitis [12]

There have been several reports of acute pancreatitis related to colchicine; including accidental ingestion of a plant (*Colchicum autumnale*) thought to be wild garlic [3], intraurethral administration of colchicine for condyloma acuminata [5], and after intentional oral overdoses of colchicine [6,7]. These patients had severe colchicine toxicity associated with multi-organ failure and death in one case [7].

Patients with renal impairment have reduced colchicine clearance, may be more susceptible to colchicine toxicity and require cautious dosing [1,2]. In this case report, acute pancreatitis occurred in an elderly man with pre-existing renal impairment after two days of oral colchicine 1 mg daily for gout in the big toe. Unlike with colchicine overdose-related pancreatitis [5-7], this patient did not experience severe colchicine toxicity, myelosuppression or deteriorating multi-organ dysfunction. Clinicians need to be cautious when prescribing colchicine in patients with renal impairment [1,2] as isolated acute pancreatitis (or potentially even more severe toxicity) may occur in these patients even with therapeutic doses of colchicine.

## Competing interests

The author(s) declare that they have no competing interests.

## Authors' contributions

JYST was fully involved in writing, reviewing and approving the manuscript.
